# Birth-related, medical, and diagnostic characteristics in younger versus older children with avoidant/restrictive food intake disorder (ARFID)

**DOI:** 10.1186/s40337-023-00908-7

**Published:** 2023-10-26

**Authors:** Luise Brosig, Dominik Düplois, Andreas Hiemisch, Wieland Kiess, Anja Hilbert, Franziska Schlensog-Schuster, Ricarda Schmidt

**Affiliations:** 1 Behavioral Medicine Research Unit, Department of Psychosomatic Medicine and Psychotherapy, Integrated Research and Treatment Center Adiposity Diseases, Leipzig University Medical Center, Stephanstrasse 9a, 04103 Leipzig, Germany; 2 LIFE Leipzig Research Center for Civilization Diseases, Leipzig University, Philipp-Rosenthal-Strasse 27, 04103 Leipzig, Germany; 3 Hospital for Children and Adolescents, Center for Pediatric Research, Leipzig University Medical Center, Liebigstrasse 20a, 04103 Leipzig, Germany; 4 Department of Child and Adolescent Psychiatry, Psychotherapy and Psychosomatics, Leipzig University Medical Center, Liebigstrasse 20a, 04103 Leipzig, Germany; 5 University Hospital of Child and Adolescent Psychiatry and Psychotherapy, University of Bern, Bern, Switzerland

**Keywords:** Avoidant/restrictive food intake disorder, Age, Comparison, Comorbidities, Perinatal complications

## Abstract

**Background:**

Although avoidant/restrictive food intake disorder (ARFID) presents the replacement and extension of feeding disorders of infancy and childhood, previous research into ARFID concentrated mainly on older patients. While birth-related characteristics play an etiologic role in feeding disorders, virtually nothing is known so far in ARFID. Therefore, the first aim of the study was to identify differences in birth-related characteristics in younger vs. older children with ARFID. Second, differences in physical and mental comorbidities, and third, diagnostic features between age groups were analysed.

**Methods:**

Among *N* = 51 in- and outpatient treatment-seeking patients, *n* = 23 patients aged 0–5 years (30% girls) and *n* = 28 patients aged 6–17 years (57% girls), with an interview-based diagnosis of ARFID were included. Data on the pre- and perinatal period and mental and physical comorbidities were derived from patients’ medical records, while diagnostic criteria, main ARFID presentation, and sociodemographic variables were collected through diagnostic interview.

**Results:**

Significantly, younger patients with ARFID were born more often preterm and had more pre- and perinatal complications and a higher incidence of postnatal invasive procedures. Patients with ARFID aged 0–5 years presented significantly more physical comorbidities and conditions, especially congenital anomalies, while mental comorbidities, especially mood disorders, were significantly more common in patients with ARFID aged 6–17 years. No age differences were found for the distribution of diagnostic criteria and main ARFID presentation.

**Conclusion:**

This is the first study which aimed to identify age-specific characteristics in patients with ARFID with potential relevance for diagnosis and treatment. Especially birth-related complications, including invasive procedures postnatally, may be associated with developing ARFID, highlighting the importance of a closer view on these potential risk factors of the disorder. Future research with longitudinal design and larger samples may allow more detailed information on further age-specific associations, symptom trajectories, and age-specific risk factors for ARFID.

**Supplementary Information:**

The online version contains supplementary material available at 10.1186/s40337-023-00908-7.

## Abstract (plain language summary)

Avoidant/restrictive food intake disorder (ARFID) is a feeding and eating disorder characterized by a highly limited amount and/or variety of food intake accompanied by weight loss or reduced growth, nutritional deficiencies, the dependency on enteral nutrition or oral nutritional supplements, or psychosocial impairment. Although the knowledge about ARFID is currently expanding, there is still a lack of information whether the disorder presents differentially among younger and older youths. The present study examined n = 23 children aged 0–5 years in comparison to n = 28 patients aged 6–17 years in birth-related, medical, and diagnostic features. ARFID was assessed by clinical interview and questionnaire data, and medical records were used to derive clinical information. While younger patients with ARFID were more likely to be born preterm, had complications after birth and more co-occurring physical diseases, older patients with ARFID showed more mental illnesses. These findings underline the relevance of further investigations on age-dependent characteristics of ARFID to adapt diagnostic assessment and treatment of the disorder. No significant age differences were found for diagnostic criteria and presentations of ARFID, indicating that these features are applicable for patients of all child ages.

**Supplementary Information:**

The online version contains supplementary material available at 10.1186/s40337-023-00908-7.

## Introduction

Avoidant/restrictive eating disorder (ARFID; 1) is characterized by a highly limited amount and/or variety of food intake, accompanied by weight loss or reduced growth (diagnostic criterion A1), nutritional deficiencies (A2), the dependency on enteral nutrition or oral nutritional supplements (A3), or psychosocial impairment (A4; 1). Food intake is not motivated by body image disturbances, but driven by sensory sensitivities, fear of aversive consequences of eating, or lack of interest, for example [2]. Previous research on ARFID showed a prevalence up to 23% in treatment-seeking adolescents with an eating disorder [3–7] and up to 64% in treatment-seeking children with a feeding disorder [8, 9]. Despite the high prevalence in younger patients and the fact that ARFID replaced and extended the feeding disorder of infancy and early childhood for children under 6 years [10], emerging research on ARFID concentrated on patients between the age of 6–18 years [3, 11]. Yet, there is evidence that patients with ARFID may differ in certain clinical characteristics depending on their age—which could be important for the identification of the disorder and its treatment.

Etiologically, researchers have theorized that abnormalities in taste perception, homeostatic appetite, and fear responsiveness may underlie ARFID [2], beyond early adverse experiences including parental pressure to eat and traumatic eating events [12] or genetic components in those with sensory sensitivities [13]. Notably, due to ARFID’s diagnostic origin [1, 10], it is plausible that patients with ARFID show similar etiological features as patients with feeding disorders, at least in younger patients. For example, pre- and perinatal complications, including prematurity and being small for gestational age (SGA), are known to be risk factors for developing feeding difficulties and disorders [14–18] as well as anorexia nervosa (AN) and bulimia nervosa [19, 20], but virtually nothing is known about these conditions in ARFID. First data on birth-related characteristics in children aged 0–10 years with ARFID have been reported by Krom et al. [8] showing a rate of 35% preterm births, 19% of patients being SGA, and having a median birth weight of 2700 g, indicating strong deviations from international data on birth-related characteristics [21]. Complications during pregnancy, like gestational diabetes, preeclampsia, or eclampsia increased the risk of feeding or eating disorders other than ARFID [19, 20, 22], similar to invasive procedures after birth, such as nasogastric or endotracheal tube insertions [23–26].

Clinically, patients with ARFID were found to have substantial mental comorbidity, especially anxiety and depressive disorders [11, 27–29] in older children (aged 10–18 years; 30, 31), while evidence in younger children remains scarce. In younger samples with ARFID, neurodevelopmental disorders, like autism spectrum disorder [1], were common [32–35], which goes along with findings from feeding disorder research, demonstrating associations between developmental disabilities and feeding problems [15]. Somatically, 19–44% of patients with ARFID aged 0–18 years showed gastrointestinal symptoms and disorders [11]. Other co-occurring medical diagnoses like endocrine, neurological, or oncological conditions, low bone density, or electrolyte abnormalities have also been identified, with research focusing mainly on patients older than 5 years [7, 29, 36, 37]. However, based on a high prevalence of 90% of concurrent diseases in patients with ARFID aged 0–10 years [8], especially diseases of the digestive system, respiratory diseases, and congenital malformations, physical comorbidities and conditions may also play an important role in younger patients.

Concerning the prevalence of diagnostic criteria A1–A4 associated with avoidant-restrictive food intake, only limited evidence is available overall and specifically for younger patients with ARFID. For ARFID presentations, sensory-based food avoidance seemed to be a common driver across ages in population-based studies and studies including treatment-seeking children and adolescents > 4 years [28, 32, 38, 39]. Evidence from research on feeding disorders [40] strengthens the assumption that also in patients with ARFID, sensory sensitivities towards food and lack of interest in eating may develop early in life and are longstanding [2, 32, 41, 42], while eating- or food-related anxiety may develop across ages [1].

In addition to recently proposed neurobiological theoretical frameworks on the development of ARFID, this study focused on an early exposure model investigating the presence of adverse birth-related and medical characteristics in ARFID. To increase awareness of age-specific characteristics and examine potentially etiologically relevant factors in ARFID, the primary aim of the study was to identify differences in birth-related characteristics between age groups of 0–5, representing the former age range for feeding disorders, and 6–17 years. Younger patients were expected to have an earlier week of birth, to be more often preterm and SGA, to have a lower weight and length of birth, and to show more complications of the pre- and perinatal period and early invasive procedures or surgery than older patients. The second aim of the study was to examine age differences in physical and mental comorbidity in ARFID, hypothesizing a higher rate of physical comorbidities and conditions as well as autism spectrum disorder and neurodevelopmental disorders in the younger versus older age group, whereas depressive and anxiety disorders were suggested to predominate in older patients with ARFID. As a third aim, the age-specific distribution of diagnostic criteria and ARFID’s main presentation was examined exploratively.

## Methods

### Participants and procedure

The sample was recruited at the Eating and Feeding Disorder Unit of Leipzig University Medical Centre, Germany, offering inpatient and outpatient treatment for children and adolescents with feeding and eating disorders as part of a larger study on the evaluation of the ARFID module for the Eating Disorder Examination [43] between February 2018 and October 2021. Inclusion criteria for the present study were an interview-based diagnosis of ARFID and an age between 0 and 17 years. There were no exclusion criteria except for insufficient German language skills, which were necessary to perform the diagnostic assessment. Parents of children aged 0–17 years gave written informed consent and children ≥ 8 years provided written informed assent prior to participation. The Ethics Committee of the Medical Faculty of Leipzig University, Germany, approved the study. All families were offered 15 € for their participation.

Out of 113 patients with restrictive feeding or eating behaviour being eligible, *n* = 35 (31.0%) did not meet criteria for ARFID, *n* = 12 (10.6%) rejected participation, *n* = 12 (10.6%) could not be reached, *n* = 2 (1.8) were excluded due to insufficient German language skills, and *n* = 1 (0.9%) did not complete the full diagnostic interview. The final sample consisted of *N* = 51 treatment-seeking children and adolescents with an ARFID diagnosis.

### Measures

#### ARFID module for the Eating Disorder Examination

The ARFID module [44] for the (Ch)EDE [45, 46] was used for diagnosis of ARFID according to the DSM-5 [1] and collection of clinical characteristics. The child and adolescent version of the EDE ARFID module with good reliability and validity [43, 44] was performed by trained and regularly supervised research assistants at the end of the full-length (Ch)EDE, whereas the parent version of the ARFID module was used stand-alone. All diagnostic assessments were discussed in a multiprofessional team including a clinical psychologist (PhD-level) with longstanding experience in the assessment of eating disorders. As part of the clinical interview, ARFID diagnostic criteria, including significant weight loss (A1), nutritional deficiency (A2), dependence on enteral feeding or oral nutritional supplements (A3), and marked interference with psychosocial functioning (A4) were evaluated [1]. In addition, information on age of illness onset in months and the presence of six different presentations of ARFID were evaluated including lack of interest in eating food, sensory sensitivity, fear of aversive consequences, emotional problems, physical problems, and ritualized eating behaviour. For all patients who met the criteria for more than one presentation, two different raters determined a main presentation based on parent and child report independently.

#### Anthropometric and sociodemographic variables

Height (cm) and body weight (kg) were objectively measured at the time of the interview and used to calculate body mass index-standard deviation scores (BMI-SDS) based on age- and sex-specific reference data from Germany [47]. Patients’ age, sex, and nationality as well as parental age, sex, BMI (kg/m^2^), education (≥ 12 school years, < 12 school years), and marital status (single, married, separated, widowed) were based on parent report and assessed to characterize the study sample.

#### Birth-related characteristics

Birth-related characteristics included information about birth week, anthropometry (birth weight in gram and length in cm), complications during pregnancy (e.g., gestational diabetes, gestational hypertension, preeclampsia, eclampsia, polyhydramnios), during birth (e.g., unscheduled C-Section, intrauterine hypoxia, umbilical cord wrap, preterm placenta solution), or after birth (e.g., adaptive disorder, respiratory distress, necessary ventilation, operation immediately after birth), the presence of C-Section, and early invasive procedures (e.g., gastric tube, invasive ventilation, insert of a gastrostomy, thoracotomy, laparotomy). The need for early invasive procedure or surgery was rated following the definition for invasive procedures of Cousins et al. [48]. Since the youngest patient was 5 months old, only procedures or surgery in the first 4 months after birth were included, to enable comparability across the different age groups.

All patients born before 37 completed weeks of gestation were coded as preterm birth following the World Health Organisation (WHO) definition [49]. Patients whose birth weight was < 10th percentile for gestational age were classified as SGA. For calculating the percentiles, the Fenton growth chart [50] was used.

#### Mental and physical comorbidities and conditions

Mental comorbidities were categorized into mood (affective) disorders (F30–F39), neurotic, stress-related and somatoform disorders (F40–F48), pervasive developmental disorders (F84), and behavioural and emotional disorders with onset usually occurring in childhood and adolescence (F90–F98) based on their diagnosis made by clinicians according to criteria of the 10th revision of the International Classification of Diseases (ICD-10; 51).

Physical comorbidities and associated conditions were summarized in four categories, gastrointestinal disorders and problems, metabolic disorders, early onset respiratory distress, and congenital anomalies, based on their ICD-10 code (Additional file 1: Table S1). The categories were chosen based on their presumed importance for ARFID [1]. For congenital anomalies, only major anomalies causing significant medical intervention, social or cosmetic consequences, were included following the definition for congenital anomalies of the WHO [52].

All clinical data were derived from patients’ medical records, collected and rated through two research assistants independently.

### Data analytic plan

In order to evaluate age differences between two groups (0–5 years, 6–17 years) in birth-related characteristics, univariate analyses of variance were performed for continuous variables (week of birth, birth weight and length) and χ^2^ analyses were used for nominal variables (presence of pre- and perinatal complications, C-Sections, invasive procedures). Similarly, the association of mental and physical comorbidities and age group was analysed using χ^2^ analyses, as were the distribution of DSM-5 ARFID diagnostic criteria A1–A4 (weight loss, nutritional deficiencies, dependency on enteral nutrition/oral supplements, psychosocial impairment) and ARFID presentations (fear of aversive consequences, lack of interest, sensory sensitivity, emotional problems, ritualized eating behaviour, physical problems). For variables deviating from normal distribution and variance homogeneity, nonparametric tests (e.g., Mann–Whitney *U* tests) were conducted, but the results were only reported when deviating from parametrical tests.

Effect sizes were estimated with Cohen’s *d* and Cramér’s *V*, which can be interpreted as small (0.2 ≤ *d* < 0.5 or 0.1 ≤ *V* < 0.3), medium (0.5 ≤ *d* < 0.8 or .3 ≤ *V* < 0.5), or large (*d* ≥ 0.8 or *V* ≥ 0.5). For all statistical analyses IBM^®^ SPSS Statistics^®^ version 27.0 was used with a two-tailed α < 0.05.

## Results

### Sample description

The final sample consisted of *N* = 51 inpatient (*n* = 26, 51%) and outpatient (*n* = 25, 49%) treatment-seeking children and adolescents with ARFID based on age groups 0–5 years (*n* = 23, 45%) and 6–17 years (*n* = 28, 55%). The sample had a mean age of 7.49 ± 5.44 years, was balanced for sex (*n* = 23 girls, 45%), and had a mean BMI-SDS of − 1.53 ± 1.02, with *n* = 21 (41%) having severe underweight (BMI-SDS ≤ − 1.88), *n* = 12 (24%) underweight (− 1.88 < BMI-SDS ≤ − 1.28), and *n* = 18 (35%) having normal weight (− 1.28 < BMI-SDS < 1.28) (Table 1).

**Table 1:** Sociodemographic and anthropometric characteristics of the sample

	Total sample (*n* = 51)	Age group 0–5 years (*n* = 23)	Age group 6–17 years (*n* = 28)	Test	*p*	*|ES|*
Child sociodemographics						
Age, years, *M* (*SD*)	7.49 (5.44)	2.35 (1.82)	11.71 (3.33)	*t*(49) = − 12.065	< 0.001	3.396
Sex, female, *n* (%)	23 (45.10)	7 (30.40)	16 (57.10)	Fisher’s exact test	0.090	0.267
Nationality, German, *n* (%)^a^	43 (100.00)	18 (100.00)	25 (100.00)	–	–	–
Child anthropometrics (objective), *M* (*SD*)						
Height, cm	120.00 (34.29)	88.73 (17.44)	145.68 (20.31)	*t*(49) = − 10.610	< 0.001	2.986
Weight, kg	23.86 (14.78)	11.87 (5.19)	33.71 (12.63)	*t*(37.32) = − 8.333	< 0.001	2.184
BMI-SDS	− 1.53 (1.02)	− 1.25 (1.11)	− 1.76 (0.89)	*t*(49) = 1.812	0.076	0.513
Child weight status, *n* (%)^b^				χ^2^(2) = 4.297	0.117	0.290
Severe underweight	21 (41.20)	6 (26.10)	15 (53.60)			
Underweight	12 (23.50)	6 (26.10)	6 (21.40)			
Normal weight	18 (35.3)	11 (47.80)	7 (25.00)			
Child treatment-setting status, *n* (%)				Fisher’s exact test	0.404	0.136
Inpatient treatment	26 (51.00)	10 (43.50)	16 (57.10)			
Outpatient treatment	25 (49.00)	13 (56.50)	12 (42.90)			
Parent sociodemographics						
Age, years, *M* (*SD*)	37.76 (7.01)	33.24 (3.72)	40.96 (7.07)	*t*(36.46) = − 4.537	< 0.001	1.329
Sex, female, *n* (%)^a^	38 (74.50)	17 (100.00)	21 (87.50)	Fisher’s exact test	0.254	0.236
High education (≥ 12 school years), *n* (%)^a^	21 (51.22)	8 (47.10)	13 (54.20)	Fisher’s exact test	0.756	0.070
Marital status, *n* (%)^a^				χ^2^(2) = 0.437	0.804	0.103
Single	10 (24.39)	5 (29.40)	5 (20.80)			
Married	28 (68.29)	11 (64.70)	17 (70.80)			
Separated	3 (7.31)	1 (5.90)	2 (8.30)			
Widowed	0 (0.00)	0 (0.00)	0 (0.00)			
BMI (kg/m^2^, subjective), *M* (*SD*)	25.21 (6.39)	24.90 (7.04)	25.45 (6.00)	*t*(40) = − 0.268	0.790	0.085

*BMI* body mass index (kg/m^2^), *SDS* standard deviation score

^a^Due to missing data, values may not sum up to *N* = 51 (100%)

^b^Child weight status was determined by German reference data classifying severe underweight (BMI-SDS ≤ − 1.88), underweight (− 1.88 < BMI-SDS ≤ − 1.28), normal weight (− 1.28 < BMI-SDS < 1.28) [47]

### Age group comparisons

#### Birth-related characteristics

Significantly more children aged 0–5 years were born preterm (*p* < 0.05, medium effect), were more affected by postnatal complications (*p* < 0.01, medium effect), and received more invasive procedures postnatally (*p* < 0.001, large effect) compared to children aged 6–17 years. No group differences emerged for birth week, birth weight and length, being SGA and the number of C-Sections (all *p*s > 0.05, small to medium effects), see Table 2.

**Table 2:** Birth-related and medical characteristics of patients with ARFID as a function of age group

	Age group 0–5 years		Age group 6–17 years		Test	*p*	*|ES|*
	*M *(SD)	*n*	*M* (SD)	*n*			
Birth-related characteristics							
Week of birth	37.74 (3.40)	23	38.46 (3.79)	26	*F*(1, 48) = 0.649	0.424	0.199
Preterm birth (< 37 + 0) (*n*, %)	12 (52.17)	23	6 (21.40)	26	Fisher’s exact test	0.043	0.301
Birth Weight, g	2681.09 (1011.62) (Median 2895.00)	23	3094.37 (872.42) (Median 3280.00)	27	*F*(1, 48) = 2.407	0.127	0.440
Birth length, cm	47.52 (5.70) (Median 49.00)	23	48.38 (5.61) (Median 50.00)	27	*F*(1, 48) = 0.997	0.323	0.152
Small for gestational age (birth weight < 10th percentile)	7 (30.40)	23	4 (14.30)	25	Fisher’s exact test	0.311	0.172
C-section (*n*, %)	9 (50.00)	18	8 (38.10)	21	Fisher’s exact test	0.528	0.120
Complications (*n*, %)	20 (90.91)	22	15 (55.56)	27	Fisher’s exact test	0.010	0.389
Complications during pregnancy	13 (65.00)	20	9 (36.00)	25	Fisher’s exact test	0.075	0.288
Complications of birth	11 (61.11)	18	10 (38.46)	26	Fisher’s exact test	0.220	0.233
Complications postnatal	14 (82.35)	17	8 (32.00)	25	Fisher’s exact test	0.002	0.495
Invasive procedure postnatal	16 (69.57)	23	3 (11.11)	27	Fisher’s exact test	< 0.001	0.600
Medical characteristics (*n*, %)							
Mental and/or physical comorbidities/conditions	20 (86.96)	23	20 (74.07)	27	Fisher’s exact test	0.308	0.161
Mental comorbidities	3 (13.04)	23	16 (59.26)	27	Fisher’s exact test	0.001	0.475
Mood (affective) disorders (ICD: F30–39)	0 (0.00)	23	9 (33.33)	27	Fisher’s exact test	0.002	0.432
Pervasive disorders (ICD: F84)	0 (0.00)	23	1 (3.70)	27	Fisher’s exact test	0.999	0.132
Neurotic disorders (ICD: F40–48)	1 (4.35)	23	4 (14.81)	27	Fisher’s exact test	0.357	0.174
Behavioural disorders (ICD: F90–98)	2 (8.70)	23	5 (18.52)	27	Fisher’s exact test	0.430	0.141
Physical comorbidities/conditions (*n*, %)	19 (82.61)	23	10 (37.04)	27	Fisher’s exact test	0.002	0.460
Gastrointestinal diseases	6 (26.09)	23	8 (29.63)	27	Fisher’s exact test	0.999	0.039
Metabolic disorders	2 (8.70)	23	2 (7.41)	27	Fisher’s exact test	0.999	0.024
Respiratory distress	6 (26.09)	23	2 (7.41)	27	Fisher’s exact test	0.121	0.254
Congenital anomalies	12 (52.17)	23	1 (3.70)	27	Fisher’s exact test	< 0.001	0.551

*ARFID* avoidant/restrictive food intake disorder, *ICD* international classification of diseases, *C-Section* caesarean section, U, Mann–Whitney test value; *F*, F-ratio; χ^2^, Chi square test value; For effect size (ES), Cramér’s *V* or *d* was reported for categorical or continuous variables

#### Mental and physical comorbidities and conditions

The majority of patients in the younger (*n* = 20, 87%) and older (*n* = 20, 74%) age group had mental and/or physical comorbidities and conditions. Older patients showed significantly more mental comorbidity, especially mood (affective) disorders (both *p* < 0.01, medium effect) versus younger patients, while there was no difference in the occurrence of ICD-10 pervasive, neurotic, or behavioural disorders (all *p*s > 0.05, small effects).

In contrast, physical comorbidities and conditions were significantly more common in the younger than older age group (*p* < 0.01, medium effect), specifically, major congenital anomalies (*p* < 0.001, large effect, Additional file 1: Table 2). No differences were found for gastrointestinal diseases, metabolic, and respiratory disorders (all *p*s > 0.05, small to medium effects).

#### Diagnostic characteristics

As shown in Table 3, there was no statistically significant difference in the distribution of the DSM-5 diagnostic criteria A1–A4 (*p* > 0.05, medium effect). In both age groups, most patients fulfilled the diagnostic criterion A1 (83% vs. 93%). While criterion A2 was the least occurring criterion in younger patients (35%), criterion A3 was least met in older patients (46%).

**Table 3:** Explorative analysis of diagnostic characteristics of patients with ARFID as a function of age group

	Age group 0–5 years		Age group 6–17 years		Test	*p*	*|ES|*
	*M* (SD)	*n*	*M* (SD)	*n*			
DSM-5 diagnostic criteria (*n*, %)							
A1 weight loss or reduced growth	19 (82.61)	23	26 (92.86)	28	Fisher’s exact test	0.390	0.158
A2 nutritional deficiencies	8 (34.78)	23	16 (57.14)	28	Fisher’s exact test	0.160	0.223
A3 dependency on enteral nutrition or food supplementation	17 (73.91)	23	13 (46.43)	28	Fisher’s exact test	0.085	0.278
A4 psychosocial impairment	9 (39.13)	23	18 (64.29)	28	Fisher’s exact test	0.095	0.251
Main ARFID presentation (*n*, %)					χ^2^ (5) = 7.820	0.098	0.395
Fear of aversive consequences	5 (22.73)	22	3 (10.71)	28			
Lack of interest	5 (22.73)	22	3 (10.71)	28			
Sensory sensitivity	7 (31.81)	22	12 (42.86)	28			
Emotional problems	1 (4.55)	22	8 (28.57)	28			
Ritualized eating behaviour	0 (0.00)	22	0 (0.00)	28			
Physical problems	6 (27.27)	22	2 (7.14)	28			

*ARFID* avoidant/restrictive food intake disorder, *DSM-5* Diagnostic and Statistical Manual of Mental Disorders

Age groups differed by trend in the distribution of ARFID’s main presentation (*p* < 0.10, medium effect). Sensory sensitivity was most common in both groups (32% vs. 43%). Physical problems were more likely to occur in younger patients (27% vs. 7%) and emotional problems occurred more often in patients between 6 and 17 years (5% vs. 29%).

## Discussion

This interview-based study of treatment-seeking patients with ARFID is the first study examining birth-related, medical, and diagnostic characteristic as a function of patients’ age. In terms of birth-related characteristics, significantly more patients aged 0–5 years were born preterm, exposed to complications of the pre- and perinatal period, and received invasive procedures postnatally compared to patients aged 6–17 years. Regarding mental and physical comorbidities, younger patients showed significantly more physical comorbidities and conditions, especially congenital anomalies, while mental comorbidities, in particular depressive disorders, were significantly more common in older patients. Exploratively examined age differences in ARFID diagnostic criteria and main presentation indicated medium-sized differences, which may be relevant for assessment and treatment.

Although previous feeding and eating disorder research underlined the relevance of associations between birth-related characteristics and the development of feeding and eating problems at early [14, 15, 17, 18] and later ages [19, 20], there was a lack of research on age-specific associations to ARFID so far. In line with hypothesis, a significantly higher rate of patients born preterm were found in the younger than older age group, indicating that prematurity may be a risk factor for developing ARFID at an early age. A reason for this may be the relation to a significantly higher rate of complications in the pre- and perinatal period, especially postnatal, in younger patients with ARFID. Furthermore, eating is a complex neurodevelopemental and interactional process including sucking, swallowing, breathing and feeding coordination [53]. Previous work already showed that the integration of these skills is significantly delayed in children born preterm and that these children continue to show high rates of oral-motor eating difficulties and behavioral eating problems [18, 53–55]. Together with the finding of Walton et al. [18] that mealtimes with preterm born infants are characterized by parental anxiety and coercive, negative feeding interactions, it seems possible that children born preterm may have a higher vulnerability to develop a disorder like ARFID in early childhood.

The tendency for having early onset respiratory distress more often (26% vs. 7%, medium effect) may partly explain the significantly higher rate of invasive procedures after birth in younger than older patients (70% vs. 11%). These high rates indicate that invasive procedures, such as nasogastric feeding, invasive ventilation or tube placement, which are known to be risk factors for developing later eating problems, such as chronic feeding problems, refusal to eat, vomiting and swallowing problems or facial defensive behaviour [14, 23–25, 56], could also play an important role in the development of early-onset ARFID. In the research on feeding disorders, these interventions contributed negatively to the development of oral feeding skills [14], for example by linking negative oral stimuli to food intake [57], which could lead into food aversion as a protective mechanism as described by Chatoor et al. [40] and others [24]. The same processes potentially play a role in the development of ARFID, leading to the need for further research on the links between birth-related complications or invasive interventions and the development of ARFID and its various manifestations.

Against our hypothesis, there were no age differences in gestational age, birth weight and length, and the number of C-Sections (small to medium effects), which may be related to the fact that both the younger and older age group were characterized by lower gestational age (37.7 and 38.5 vs. 39.8 weeks; 21), more preterm births (52% and 21% vs. 12%; 58), lower birth weight (2895 g and 3280 g vs. 3480 g; 21), and a higher number of C-Sections (50% and 38% vs. 31%; 59) than German reference data. Deviations in birth-related characteristics from the population may be likely due to a higher rate of pre- and perinatal complications or congenital anomalies in patients with ARFID than those from the population [21], although other possible confounders for birth-related outcomes, such as maternal anthropometry and parity, remain to be investigated in patients with ARFID. Overall, the present results indicate that especially complications of the pre- and perinatal period and resulting procedures rather than birth anthropometrics itself may impact an early development of ARFID.

In terms of the total sample, the results on gestational age (38.1 weeks), prematurity (37%), birth weight (2904 g), and being SGA (23%) in the present study were highly comparable to those found in the only study reporting information on birth-related characteristics in *n* = 48 0–10-year-old Dutch children with ARFID (gestational age 38.1 weeks, prematurity 35%, birth weight 2700 g, SGA 19%; [8]), which likely signals reliable data. Considering child ages 0–5 years only, somewhat higher rates for being preterm birth (52% vs. 35%) and SGA (30% vs. 19%) were found relative to the sample by Krom et al. [8], which might be due to the higher percentage of congenital anomalies (52% vs. 23%).

In line with hypothesis and previous research [11], all patients showed a high rate of mental and/or physical comorbidities and conditions (87% and 74%), whereby mental comorbidities were typical for older patients with ARFID (59%). Following the work from Katzman et al. [31] and Duncombe Lowe et al. [30], who found rates of 49% and 57% anxiety disorders in older children with ARFID and increasing numbers of comorbid depression with higher age, anxiety and depressive disorders were frequently assigned diagnoses in patients aged 6–17 years (15 and 33%). Contrasting previous research demonstrating associations between neurodevelopemental disorders and ARFID in treatment-seeking samples with eating and feeding disorders [11, 32, 34], there was only one patient with a comorbid autism spectrum disorder in the whole sample, without significant differences and only small effects between age groups in other mental disorders. Notably, the prevalence of mental disorders in younger children with ARFID might be generally underrestimated due to difficulties in assessment and diagnostic classification of mental disorder in early childhood per se [60].

Regarding physical comorbidity, the present results aligned with hypothesis and Krom et al. [8] who showed that 90% of children with ARFID aged 0–10 years presented with an additional somatic disease. In the present study, more than 80% of the younger age group showed physical comorbidities and associated conditions, with major congenital anomalies, like deformations of the cleft lip and palate, congenital anomalies of the circulatory or digestive system, or chromosomal abnormalities occurring in 52% of patients aged 0–5 years, which is in line with studies showing feeding and swallowing problems, for example, pharyngeal phase dysphagia, in young children with Down syndrome [61, 62]. Gastrointestinal diseases were relatively common across ages (26 vs. 30%) in accordance with the findings of Sanchez-Cerezo et al. [11] showing that 19–44% of all patients with ARFID had gastrointestinal symptoms or disorders.

ARFID’s diagnostic criteria were examined exploratively due to lacking evidence on age-specific distributions. In a previous study, Krom et al. [8] showed that *N* = 64 patients between 0 and 10 years with ARFID most often met the criterion for tube feeding or oral supplements (A3, 74%), while weight loss was less common (A1, 9%), whereas in *N* = 207 treatment-seeking children with ARFID between 5 and 18 years, criteria A1 (90%) and A4 (67%) were very common [31], consistent to findings in other studies [39, 63–65]. The medium-sized effects in diagnostic criteria found in our patients may point to clinically relevant age differences, but are subject to replication. Criterion A1 was most common in both age groups (83% vs. 93%) consistent with previous findings of treatment-seeking samples [8, 31]. Nutritional deficiencies (A2) occurred more often in older children (35% vs. 57%, medium effect), which could be explained by a rather longer duration of illness, leading into depletion of vitamin- and nutrient stores. The fact that younger patients were more likely to depend on enteral nutrition (A3) than older patients (74% vs. 46%, medium effect) may counteract nutritional deficiencies and weight loss and is consistent with findings by Katzman et al. [31], where criterion A3 was met more often by younger (5–9 years) versus older (15–18 years) patients. Concerning psychosocial impairment (A4), this criterion was descriptively more often met by older than younger patients (39% vs. 64%, medium effect), which is consistent with our finding of more mental comorbidities in older patients with ARFID.

In line with associated comorbidities, age groups differed with medium effect in the distribution of the main ARFID presentation. Physical problems appeared more often in younger (27% vs. 7%), while emotional problems were more typical for older patients with ARFID (5% vs. 29%). Sensory sensitivity was the most common presentation in patients aged 0–5 years (32%) and patients aged 6–17 years (43%), consistent with previous research [28, 32, 38]. Similary, fear of aversive consequences of eating and lack of interest occurred across ages, without age differences being observed. Borrowing evidence from research on feeding disorders [40], there may be similar phenotypes for low food intake in children < 6 years, just named differentially, including sensory food aversion, infantile anorexia, or posttraumatic feeding disorder. Chatoor et al. [40] highlighted that while sensory food aversions and infantile anorexia become evident during the first 3 years of life, the posttraumatic feeding disorder can be seen at any age. To support the assumption that these characteristics are similiar in patients with ARFID, further research on the genesis and time of onset of the different presentations is required.

The strengths of this study include the consideration of patients with ARFID younger than 6 years following the etiological origin of the disorder. Furthermore, objectively assessed anthropometrics, the interview-based diagnosis, and homogenuous recruitment deserve mention. Nevertheless, the sample size of *N* = 51 patients with ARFID was powered to detect large-sized effects only. The lacking ethnical diversity of the sample could limit the transferability to other samples. Similarly, findings may not generalize to non-treatment-seeking samples with ARFID. Although assessed in a standardized manner by clinicians, pre- and perinatal characteristics, comorbidities, and complications were only based on chart reviews, lacking on information about accuracy. Notably, 47% of the age group 6–17 years showed an illness onset before 6 years of age, which may have attenuated potential age differences. Explorative analyses between patients with early (< 6 years, *n* = 39) and late ARFID onset (≥ 6 years, *n* = 12) showed that the results were relatively robust. In line with results on age-group differences, the comparison between early and late ARFID onset showed that patients with an early onset had statistically significant more congenital anomalies (34% vs. 0%), postnatal complications (63% vs. 20%), and invasive procedures postnatally (47% vs. 8%), while patients with a late ARFID onset showed more mental (21% vs. 92%), especially depressive disorders (5% vs. 58%; Additional file 1: Tables 3 and 4).

This study identified age-specific characteristics for patients with ARFID including prematurity, complications of the pre- and perinatal period, invasive procedure postnatally, and mental and physical comorbidities and associated conditions. Our findings support the applicability of ARFID over the lifespan, but suggest a differential clinical characterization of patients at different ages [66]. Scientifically, the present results warrant replication in larger clinical and non-clinical samples with ARFID, ideally using a prospective design in exposed (children with birth-related complications) and non-exposed children in order to evaluate the course of ARFID symptoms over childhood and adolescence. In order to increase the understanding of the heterogeneity in ARFID presentations, future studies are recommended to examine birth-related and medical characteristics in ARFID based on ARFID’s phenotype (e.g., sensory sensitivity, fear of aversive consequences, lack of interest) as well. Longitudinal research should also adress the question which risk factors may be related to early-onset and later-onset ARFID across childhood. Although early adverse events may play a pivotal role for ARFID, it must be clarified which mechanisms are relevant in ARFID’s pathogenesis or may act as protective factors, for example, parental feeding patterns, children’s temperament, or neurobiological features. Clinically, it would be useful to monitor patients with ARFID in the long-term, in order to identify symptom and comorbidity changes having in mind that physical problems may be typical in younger and mental disorders in older patients with ARFID. Birth-related complications may be associated with developing ARFID, highlighting the importance of prevention efforts through early parental education on potential feeding problems of their children and a close follow-up on children’s feeding behaviour.

### Supplementary Information

**Additional file 1**. Supplemental Tables.

## Data Availability

The data are available from the corresponding author upon reasonable request.
