# Supplementary material for: Birth-related, medical, and diagnostic characteristics in younger versus older children with avoidant/restrictive food intake disorder (ARFID)
Source: J Eat Disord. 2023 Oct 26;11:190. doi: 10.1186/s40337-023-00908-7 (PMC10601262; doi:10.1186/s40337-023-00908-7)
Supplement: Supplementary file 1 — Additional file 1. Supplemental Tables. [file 40337_2023_908_MOESM1_ESM.docx]

**Birth-related, medical, and diagnostic characteristics in younger versus older children with avoidant/restrictive food intake disorder (ARFID)**

**by Brosig et al.**

**Supplemental Tables**

**Table 1.** Somatic comorbidities

**Table 2.** Assigned diagnoses of major congenital anomalies in the total sample

**Table 3.** Birth related and medical characteristics of patients with ARFID as a function of early (< 6 years) or late (≥ 6 years) ARFID onset

**Table 4.** Explorative analysis of diagnostic characteristics of patients with ARFID as a function of early (< 6 years) or late (≥ 6 years) ARFID onset

**Table 1**

*Categories of somatic comorbidities according to ICD-10 being classified*

| **Category** | **Gastrointestinal diseases and problems** | **Metabolic disorders** | **Early onset respiratory distress** | **Congenital anomalies** |
| --- | --- | --- | --- | --- |
| **ICD-10 diagnoses** | XI Diseases of the ingestive system (K)  I Intestinal infectious diseases (A0 – 9)  XVIII Symptoms and signs involving the digestive system and abdomen (R10 – R19) | IV Metabolic Disorders (E70 – E90) | XVI Respiratory and cardiovascular disorders specific to the perinatal period (P20 – P29) | XVII Congenital malformations, deformations and chromosomal abnormalities (Q00 – 99) |

*Note:*ICD, International Classification of Diseases.

**Table 2**

*Assigned diagnoses of major congenital anomalies in the total sample*

| **Category** | **Major congenital anomalies** |
| --- | --- |
| **ICD-10 diagnoses** | Q21.0 Ventricular septal defect  Q21.1 Atrial septal defect  Q21.3 Tetralogy of Fallot  Q24.8 Other specified congenital malformations of heart  Q25.1 Coarctation of aorta  Q35.5 Cleft hard palate with cleft soft palate  Q39.0 Atresia of oesophagus without fistula  Q39.1 Atresia of oesophagus with tracheo-oesophageal fistula  Q39.3 Congenital stenosis and stricture of oesophagus  Q41.0 Congenital absence, atresia and stenosis of duodenum  Q42.0 Congenital absence, atresia and stenosis of rectum with fistula  Q42.3 Congenital absence,atresia and stenosis of anus without fistula  Q43.3 Congenital malformations of intestinal fixation  Q45.1 Annular pancreas  Q75.4 Mandibulofacial dysostosis  Q79.2 Exomphalos  Q90.0 Trisomy 21, meiotic nondisjunction  Q93.0 Whole chromosome monosomy, meiotic nondisjunction  Q93.5 Other deletions of part of a chromosome |

*Note:* ICD, International Classification of Diseases.

**Table 3**

*Birth related and medical characteristics of patients with ARFID as a function of* *early (< 6 years) or late (≥ 6 years) ARFID onset*

|  | Early ARFID onset  (*N* = 39) |  | Late ARFID onset  (*N* = 12) |  |  | Statistical analysis | | |
| --- | --- | --- | --- | --- | --- | --- | --- | --- |
|  | *M (SD)* | *n* | *M (SD)* | *n* |  | Test | *p* | *\| ES \|* |
| Pre- and perinatal characteristics |  |  |  |  |  |  |  |  |
| Week of birth | 37.92 (3.66) | 38 | 38.82 (3.43) | 11 |  | *F*(1, 47) *=* 0.527 | .471 | 0.249 |
| Preterm birth (<37+0) (*n*, %) | 15 (39.47) | 38 | 3 (27.27) | 11 |  | Fisher’s exact test | .724 | 0.106 |
| Birth weight, g | 2822.47 (986.22) | 38 | 3163.25 (818.98) | 12 |  | *F*(1, 48) = 1.172 | .284 | 0.359 |
| Birth length, cm | 47.92 (6.04) | 38 | 49.83 (3.81) | 12 |  | *F*(1, 48) *=* 1.061 | .308 | 0.341 |
| Small for gestational age (birth weight < 10^th^ percentile) | 9 (24.32) | 37 | 2 (18.18) | 11 |  | Fisher’s exact test | .999 | 0.061 |
| C-Section (*n*, %) | 14 (48.28) | 29 | 3 (30.00) | 10 |  | Fisher’s exact test | .464 | 0.161 |
| Complications (*n*, %) | 28 (75.68) | 37 | 7 (58.33) | 12 |  | Fisher’s exact test | .285 | 0.165 |
| Complications during pregnancy | 17 (50.00) | 34 | 5 (45.45) | 11 |  | Fisher’s exact test | .999 | 0.039 |
| Complications of birth | 16 (48.48) | 33 | 5 (45.45) | 11 |  | Fisher’s exact test | .999 | 0.026 |
| Complications postnatal | 20 (62.5) | 32 | 2 (20.00) | 10 |  | Fisher’s exact test | .030 | 0.362 |
| Invasive procedure postnatal | 18 (47.37) | 38 | 1 (8.33) | 12 |  | Fisher’s exact test | .018 | 0.343 |
| Medical characteristics |  |  |  |  |  |  |  |  |
| Mental and/or physical comorbidities (*n*, %) | 29 (76.32) | 38 | 11 (91.67) | 12 |  | Fisher’s exact test | .416 | 0.164 |
| Mental comorbidities (*n*, %) | 8 (21.05) | 38 | 11 (91.67) | 12 |  | Fisher’s exact test | <.001 | 0.621 |
| Mood (affective) disorders (ICD: F30 – 39) (*n*, %) | 2 (5.26) | 38 | 7 (58.33) | 12 |  | Fisher’s exact test | <.001 | 0.590 |
| Pervasive disorders (ICD: F84) (*n*, %) | 1 (2.63) | 38 | 0 (0.00) | 12 |  | Fisher’s exact test | .999 | 0.080 |
| Neurotic disorders (ICD: F40 – 48) (*n*, %) | 2 (5.26) | 38 | 3 (25.00) | 12 |  | Fisher’s exact test | .082 | 0.281 |
| Behavioral disorders (ICD: F90 – 98) (*n*, %) | 4 (10.53) | 38 | 3 (25.00) | 12 |  | Fisher’s exact test | .337 | 0.178 |
| Physical comorbidities (*n*, %) | 23 (60.53) | 38 | 6 (50.00) | 12 |  | Fisher’s exact test | .738 | 0.091 |
| Gastrointestinal diseases (*n*, %) | 9 (23.68) | 38 | 5 (41.67) | 12 |  | Fisher’s exact test | .278 | 0.171 |
| Metabolic disorders (*n*, %) | 3 (7.89) | 38 | 1 (8.33) | 12 |  | Fisher’s exact test | .999 | 0.007 |
| Respiratory distress (*n*, %) | 7 (18.42) | 38 | 1 (8.33) | 12 |  | Fisher’s exact test | .661 | 0.118 |
| Congenital anomalies (*n*, %) | 13 (34.21) | 38 | 0 (0.00) | 12 |  | Fisher’s exact test | .022 | 0.333 |

*Note:* ARFID, avoidant/restrictive food intake disorder; ICD, International Classification of Diseases; C-Section, Caesarean section; *F*, F-ratio; χ², Chi square test value. For effect size (ES), Cramér’s *V* or *d* was reported for categorical or continuous variables.

**Table 4**

*Explorative analysis of diagnostic characteristics of patients with ARFID as a function of early (< 6 years) or late (≥ 6 years) ARFID onset*

|  | Early ARFID onset  (*N* = 39) |  | Late ARFID onset  (*N* = 12) |  |  | Statistical Analysis | | |
| --- | --- | --- | --- | --- | --- | --- | --- | --- |
|  | *M (SD)* | *n* | *M (SD)* | *n* |  | Test | *p* | *\| ES \|* |
| DSM-5 Diagnostic criteria (*n*, %) |  |  |  |  |  |  |  |  |
| A1 weight loss or reduced growth | 33 (84.62) | 39 | 12 (100.00) | 12 |  | Fisher’s exact test | .315 | 0.203 |
| A2 nutritional deficiencies | 16 (41.03) | 39 | 8 (66.67) | 12 |  | Fisher’s exact test | .187 | 0.218 |
| A3 dependency on enteral nutrition or food supplementation | 22 (56.41) | 39 | 8 (66.67) | 12 |  | Fisher’s exact test | .739 | 0.088 |
| A4 psychosocial impairment | 18 (46.15) | 39 | 9 (75.00) | 12 |  | Fisher’s exact test | .105 | 0.245 |
| Main ARFID presentation |  |  |  |  |  | χ² (5) = 27.140 | <.001 | 0.737 |
| Fear of aversive consequences (*n*, %) | 6 (15.79) | 38 | 2 (16.67) | 12 |  |  |  |  |
| Lack of interest (*n*, %) | 8 (21.05) | 38 | 0 (0.00) | 12 |  |  |  |  |
| Sensory sensitivity (*n*, %) | 18 (47.37) | 38 | 1 (8.33) | 12 |  |  |  |  |
| Emotional problems (*n*, %) | 1 (2.63) | 38 | 8 (66.67) | 12 |  |  |  |  |
| Ritualized eating behavior (*n*, %) | 0 (0.00) | 38 | 0 (0.00) | 12 |  |  |  |  |
| Physical problems (*n*, %) | 5 (13.16) | 38 | 1 (8.33) | 12 |  |  |  |  |

*Note:* ARFID, avoidant/restrictive food intake disorder; DSM-5, Diagnostic and Statistical Manual of Mental Disorders; *F*, F-ratio; χ², Chi square test value. For effect size (ES), Cramér’s *V* or *d* was reported for categorical or continuous variables.
